# Viruses Previously Identified in Brazil as Belonging to HIV-1 CRF72_BF1 Represent Two Closely Related Circulating Recombinant Forms, One of Which, Designated CRF122_BF1, Is Also Circulating in Spain

**DOI:** 10.3389/fmicb.2022.863084

**Published:** 2022-05-27

**Authors:** Javier E. Cañada-García, Elena Delgado, Horacio Gil, Sonia Benito, Mónica Sánchez, Antonio Ocampo, Jorge Julio Cabrera, Celia Miralles, Elena García-Bodas, Ana Mariño, Patricia Ordóñez, María José Gude, Carmen Ezpeleta, Michael M. Thomson

**Affiliations:** ^1^HIV Biology and Variability Unit, Centro Nacional de Microbiología, Instituto de Salud Carlos III, Majadahonda, Spain; ^2^Department of Internal Medicine, Complejo Hospitalario Universitario de Vigo, Vigo, Spain; ^3^Department of Microbiology, Complejo Hospitalario Universitario de Vigo, Vigo, Spain; ^4^Microbiology and Infectology Research Group, Galicia Sur Health Research Institute (IIS Galicia Sur), SERGAS-UVIGO, Vigo, Spain; ^5^Infectious Diseases Unit, Complejo Hospitalario Universitario de Ferrol, Ferrol, Spain; ^6^Department of Microbiology, Complejo Hospitalario Universitario de Ferrol, Ferrol, Spain; ^7^Department of Microbiology, Hospital Universitario Lucus Augusti, Lugo, Spain; ^8^Department of Clinical Microbiology, Complejo Hospitalario de Navarra, Pamplona, Spain

**Keywords:** HIV-1, circulating recombinant forms, molecular epidemiology, phylogeny, phylodynamics

## Abstract

Circulating recombinant forms (CRFs) are important components of the HIV-1 pandemic. Those derived from recombination between subtype B and subsubtype F1, with 18 reported, most of them of South American origin, are among the most diverse. In this study, we identified a HIV-1 BF1 recombinant cluster that is expanding in Spain, transmitted mainly *via* heterosexual contact, which, analyzed in near full-length genomes in four viruses, exhibited a coincident BF1 mosaic structure, with 12 breakpoints, that fully coincided with that of two viruses (10BR_MG003 and 10BR_MG005) from Brazil, previously classified as CRF72_BF1. The three remaining Brazilian viruses (10BR_MG002, 10BR_MG004, and 10BR_MG008) previously identified as CRF72_BF1 exhibited mosaic structures highly similar, but not identical, to that of the Spanish viruses and to 10BR_MG003 and 10BR_MG005, with discrepant subtypes in two short genome segments, located in *pol* and gp120^env^. Based on these results, we propose that the five viruses from Brazil previously identified as CRF72_BF1 actually belong to two closely related CRFs, one comprising 10BR_MG002, 10BR_MG004, and 10BR_MG008, which keep their CRF72_BF1 designation, and the other, designated CRF122_BF1, comprising 10BR_MG003, 10BR_MG005, and the viruses of the identified Spanish cluster. Three other BF1 recombinant genomes, two from Brazil and one from Italy, previously identified as unique recombinant forms, were classified as CRF72_BF1. CRF122_BF1, but not CRF72_BF1, was associated with protease L89M substitution, which was reported to contribute to antiretroviral drug resistance. Phylodynamic analyses estimate the emergence of CRF122_BF1 in Brazil around 1987. Given their close phylogenetic relationship and similar structures, the grouping of CRF72_BF1 and CRF122_BF1 in a CRF family is proposed.

## Introduction

HIV-1 is characterized by high genetic diversity and rapid evolution, derived from elevated mutation and recombination rates. Through these mechanisms, the HIV-1 group M, the causative agent of the AIDS pandemic, has evolved into numerous circulating genetic forms, known as subtypes, of which 10 have been identified (A–D, F–H, J–L), subsubtypes (A1–A6, F1, and F2), and circulating recombinant forms (CRFs), 118 of which are currently listed in the Los Alamos HIV Sequence Database (Los Alamos National Laboratory, 2021). In addition, geographic variants and clusters, some representing substantial proportions of viruses in certain areas, have been identified through phylogenetic analyses within subtypes, subsubtypes, and CRFs (Thomson and Nájera, 2005; Delgado et al., 2015, 2019). Genetic characterization of HIV-1 variants is of public health relevance, as it allows tracking their geographic spread and estimating their population growth and the efficacy of preventive interventions (Paraskevis et al., 2016; Rife et al., 2017; Vasylyeva et al., 2020). It has also biological and clinical relevance, as different biological properties have been associated with some HIV-1 variants (Kiwanuka et al., 2008; Pérez-Álvarez et al., 2014; Kouri et al., 2015; Venner et al., 2016; Cid-Silva et al., 2018; Song et al., 2019).

The number of CRFs is increasing incessantly, due to both the continuous generation of recombinant forms where diverse HIV-1 variants meet in the same population (Nájera et al., 2002), some of which become circulating through introduction into transmission networks, and the identification of old previously undocumented CRFs. The proportion of CRFs in the HIV-1 pandemic has increased over time, representing around 17% of infections in 2010–2015 (Hemelaar et al., 2020). Among CRFs, those derived from subtype B and subsubtype F1 are among the most numerous, 18 of which have been reported in the literature, most of them originated in South America. The most widely circulating CRF from South America is CRF12_BF, which circulates at high prevalences in Argentina and Uruguay, where unique recombinant forms (URFs) related to CRF12_BF are frequently found (Thomson et al., 2000, 2002; Carr et al., 2001). Four other CRF_BFs (numbers 17, 38, 44, and 89) related to CRF12_BF, as evidenced by shared breakpoints and phylogenetic clustering, were subsequently identified in different South American countries (Ruchansky et al., 2009; Delgado et al., 2010, 2021; Aulicino et al., 2012). Due to their common ancestry and similar structures, these five CRFs and related URFs have been proposed to constitute a “family” of recombinant viruses (Thomson and Nájera, 2005; Zhang et al., 2010; Delgado et al., 2021). By contrast, Brazilian CRF_BFs (De Sá Filho et al., 2006; Guimarães et al., 2008; Sanabani et al., 2010; Pessôa et al., 2014a,b, 2016; Reis et al., 2017, 2019) and CRF66_BF (the latter found mainly in Paraguay and Paraguayans living in Spain) (Bacqué et al., 2021) are unrelated to CRF12_BF. Similarly to the viruses of the CRF12_BF family, close relations have been reported between some Brazilian CRF_BFs: CRF28_BF and CRF29_BF (De Sá Filho et al., 2006) and CRF70_BF and CRF71_BF (Pessôa et al., 2014a).

In this study, we report the spread of a BF1 cluster in Spain whose viruses exhibit a mosaic structure identical to two Brazilian viruses previously identified as CRF72_BF1 (Pessôa et al., 2014b,^2016^), which would represent a new CRF, with the three other viruses classified as CRF72_BF1 showing highly similar, but not identical, structures. We propose that viruses previously identified as CRF72_BF1 actually belong to two closely related CRFs that constitute a CRF family.

## Materials and Methods

### Samples

Plasma and whole blood samples from HIV-1-infected individuals were collected in Spain for antiretroviral drug resistance tests and for a molecular epidemiological study. The study was approved by the Committee of Research Ethics of Instituto de Salud Carlos III, Majadahonda, Madrid, Spain. It did not require written informed consent by the study participants, as it used samples and data collected as part of routine clinical practice, and patients’ data were anonymized without retaining data allowing individual identification.

### PCR Amplification and Sequencing

An ∼1.4-kb pol fragment in protease–reverse transcriptase (Pr–RT) was amplified from plasma-extracted RNA or from whole blood-extracted DNA by (RT-)PCR followed by nested PCR, as described previously (Delgado et al., 2015), and sequenced with the Sanger method using a capillary automated sequencer. Near full-length genome (NFLG) sequences were obtained for selected samples by RT-PCR/nested PCR amplification from plasma RNA in five overlapping segments and sequenced by the Sanger method, as described (Delgado et al., 2002; Sierra et al., 2005; Cañada et al., 2021). Newly derived sequences are deposited in GenBank under accessions OL982311–OL982317 and OL982320–OL982323.

### Phylogenetic Sequence Analyses

Sequences were aligned with MAFFT v7 (Katoh and Standley, 2013). Initial phylogenetic trees with all Pr–RT sequences obtained by us were constructed *via* approximate maximum likelihood with FastTree v2.1.10 (Price et al., 2010), using the general time-reversible evolutionary model with CAT approximation to account for among-site rate heterogeneity, with assessment of node support with Shimodaira–Hasegawa (SH)-like local support values (Guindon et al., 2010). Subsequent maximum likelihood (ML) trees with sequences of interest were constructed with W-IQ-Tree (Trifinopoulos et al., 2016), using the best-fit substitution model selected by the ModelFinder program (Kalyaanamoorthy et al., 2017), with assessment of node support with the ultrafast bootstrap approximation approach (Hoang et al., 2018). Trees were visualized with MEGA v7.0 (Kumar et al., 2016).

Mosaic structures were analyzed by bootscanning (Salminen et al., 1995) with SimPlot v1.3.5 (Lole et al., 1999). In these analyses, trees were constructed using the neighbor-joining method with the Kimura two-parameter model and a window width of 250 nucleotides. The subtype affiliations of recombinant segments identified with SimPlot, whose breakpoints were more precisely located in the midpoint of transitions between BF1 subtype-discriminating nucleotides (here defined as those differing between the 75% consensus sequences of subtype B and the Brazilian F1 strain), were further phylogenetically analyzed *via* ML and Bayesian inference. These analyses were performed with IQ-Tree; PhyML (Guindon et al., 2010), using the best-fit evolutionary model selected by the SMS program (Lefort et al., 2017) and node support assessment with the approximate likelihood ratio test, SH–like procedure; and MrBayes v3.2 (Ronquist et al., 2012), using the GTR + G + I substitution model, with two simultaneous independent runs and eight chains 2–5 million generations long, ensuring that both runs reached convergence, as determined by an average standard deviation of split frequencies <0.01, discarding the first 50% of the trees in the posterior distribution as burn-in. For these analyses, we used a reconstructed BF1 ancestral sequence as outgroup, considering the phylogenetic relationship between B and F subtypes (Zhu et al., 1998), obtained with IQ-Tree. The use of a reconstructed ancestral sequence as outgroup is similar to the approach used in other studies (Travers et al., 2004; Thomson and Fernández-García, 2011; Seager et al., 2014) to prevent the long-branch attraction artifact (Bergsten, 2005) that could be caused by an outgroup whose distance to the ingroup is relatively long compared with the within-ingroup distances. This artifact can result in collapse or a substantial decrease in node support of the clades of the ingroup, particularly in short genome segments.

Phylogenetic trees and alignments used for their construction have been deposited in TreeBase, with accession URL http://purl.org/phylo/treebase/phylows/study/TB2:S29595.

### Antiretroviral Drug Resistance Determination

Antiretroviral (ARV) drug resistance was analyzed with the HIVdb program of the Stanford University’s HIV Drug Resistance Database (Rhee et al., 2003; Shafer, 2006).

### Temporal and Geographic Estimations of Clade Ancestors

The time and the most probable location of the most recent common ancestor (MRCA) of the newly defined CRF were estimated using Pr–RT sequences with the Bayesian Markov chain Monte Carlo (MCMC) coalescent method implemented in BEAST v1.10.4 (Suchard et al., 2018). Before the BEAST analysis, the existence of temporal signal in the dataset was assessed with TempEst v1.5.3 (Rambaut et al., 2016). The BEAST analysis was performed using the SRD06 codon-based evolutionary model (where the third codon position is in a partition different from the first and second positions) (Shapiro et al., 2006). We also specified an uncorrelated lognormal relaxed clock and a Bayesian SkyGrid coalescent tree prior (Gill et al., 2013). The MCMCs were run for 20 million generations. The runs were performed in duplicate, and the posterior tree files were combined with LogCombiner v1.10.4. Proper mixing of the chains was assessed with Tracer v1.6, ensuring that effective sample size values of all parameters were >200. The posterior distribution of trees was summarized in a maximum clade credibility (MCC) tree with TreeAnnotator v1.10.4, after discarding 10% of the MCMC chain as burn-in. MCC trees were visualized with FigTree v1.4.2 (Rambaut).^1^ Parameter uncertainty was summarized in 95% highest posterior density (HPD) intervals.

## Results

### Identification of a HIV-1 Cluster of F1 Subsubtype in Protease–Reverse Transcriptase Propagating in Spain

**FIGURE 1:**
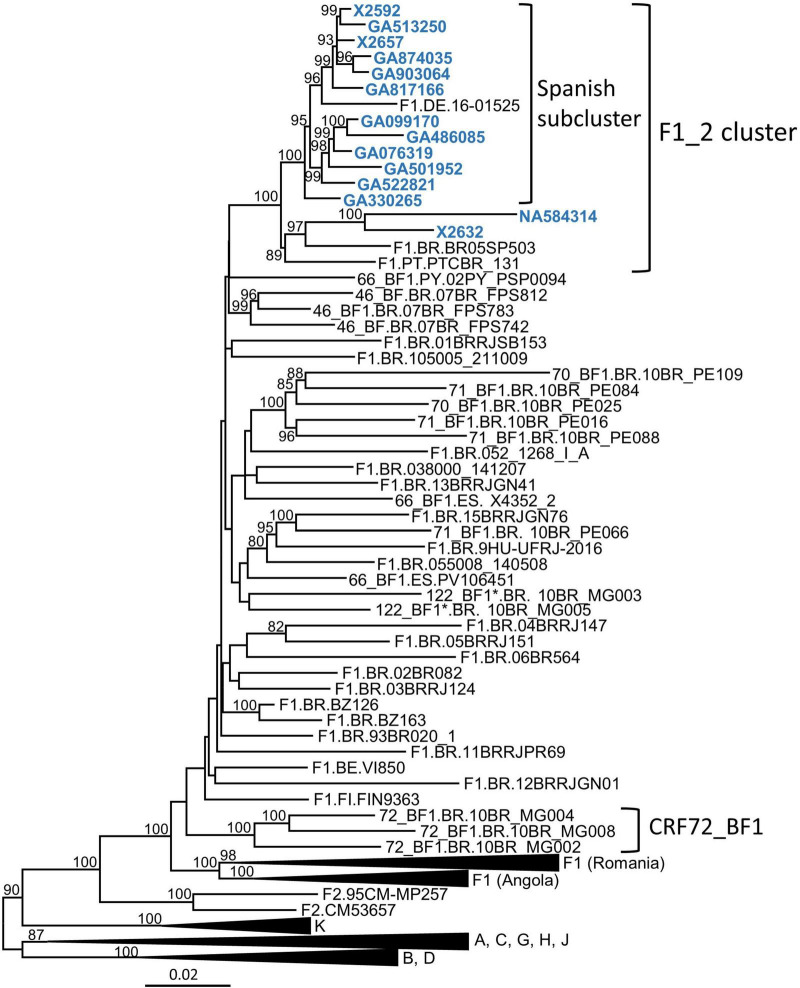
Maximum likelihood phylogenetic tree of Pr–RT sequences of the F1_2 cluster. Names of sequences obtained by us, all collected in Spain, are in blue. Only ultrafast bootstrap values ≥ 80% are shown. In database sequences, the country of sample collection is indicated before the virus name with the two-letter ISO country code: BE, Belgium; BR, Brazil, DE, Germany; ES, Spain; FI, Finland; PT, Portugal; PY, Paraguay. The scale indicates substitutions/site. *10BR_MG003 and 10BR_MG005 were originally identified as CRF72_BF1 (Pessôa et al., 2014b,^2016^), but analyses described in this study have reclassified them as CRF122_BF1.

In a molecular epidemiological study in Spain, based on Pr–RT sequences, we detected frequent grouping in clusters, several of which were of F1 subsubtype in Pr–RT (Thomson et al., 2012; Delgado et al., 2015, 2019; Gil et al., 2022). One of them, designated F1_2, which is the focus of the present study, comprised 14 individuals, 13 of them from the region of Galicia, northwest Spain (Table 1). Years of HIV-1 diagnoses were between 2007 and 2019, and transmission was predominantly heterosexual (*n* = 8), but there were three men who have sex with men (two others had non-specified sexual transmission, and no data on transmission route were available for another individual) (Table 1). Most individuals were Spanish, but three were Brazilian, one was Swiss, and one was Ukrainian. To determine whether other sequences from databases belonged to this cluster, we performed BLAST searches in the HIV Sequence Database (Los Alamos National Laboratory, 2021), incorporating the most similar sequences in the phylogenetic analyses. This allowed identifying three additional sequences that belonged to the F1_2 cluster, from Brazil, Portugal, and Germany (Figure 1). All but two of the viruses collected in Spain and the virus from Germany branched in a subcluster. Viruses from the F1_2 cluster were most closely related to viruses of the Brazilian F1 strain and to Brazilian CRF_BFs with Pr–RT derived from it.

**TABLE 1:** Epidemiological data of patients and GenBank accessions of sequences.

Sample ID	City of sample collection	Region of sample collection	Country of origin	Year of HIV diagnosis	Year of sample collection	Gender*	Transmission route*	PR–RT GenBank accession	NFLG GenBank accession
X2592	Vigo	Galicia	Spain	2008	2008	F	HT	GU326146	–
X2632	Ferrol	Galicia	Spain	2009	2009	M	MSM	GU326158	KC113006 JX140660
X2657	Vigo	Galicia	Spain	2009	2009	F	HT	GU326163	–
GA076319	Vigo	Galicia	Ukraine	2010	2010	F	HT	OL982311	–
GA099170	Vigo	Galicia	Switzerland	2011	2011	F	HT	–	OL982312
GA330265	Vigo	Galicia	Brazil	2007	2007	Trans	Sexual	OL982313	–
GA486085	Ferrol	Galicia	Spain	2018	2018	F	n.a.	–	OL982314
GA501952	Vigo	Galicia	Spain	2014	2014	F	HT	OL982315	–
GA513250	Vigo	Galicia	Spain	2012	2012	M	Sexual	OL982316	–
GA522821	Lugo	Galicia	Brazil	2019	2019	M	MSM	OL982317	–
GA817166	Vigo	Galicia	Spain	2008	2016	F	HT	OL982320	–
GA874035	Vigo	Galicia	Spain	2012	2012	M	HT	–	OL982321
GA903064	Vigo	Galicia	Spain	2012	2012	F	HT	OL982322	–
NA584314	Pamplona	Navarre	Brazil	2017	2017	M	MSM	OL982323	–

*n.a., datum not available; Trans, transgender; HT, heterosexual; MSM, man who has sex with men; sexual, unspecified sexual transmission.

### Analyses of Near Full-Length Genome Sequences

**FIGURE 2:**
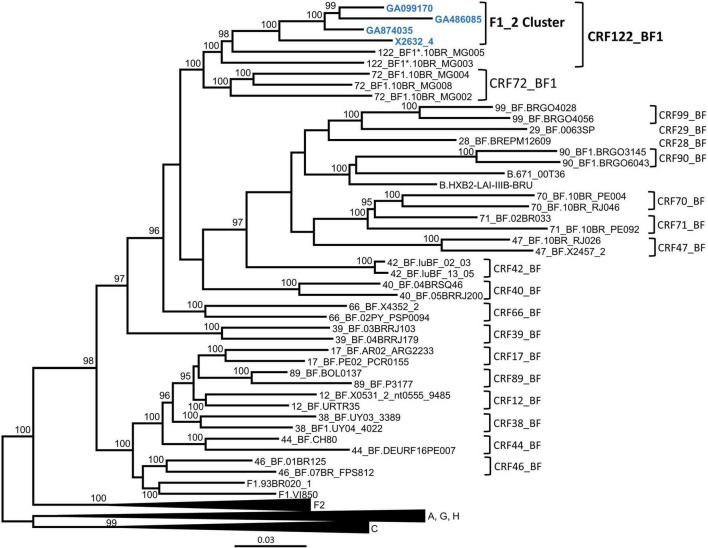
Maximum likelihood tree of NFLG sequences of viruses of the F1_2 cluster. References of published CRF_BFs and of HIV-1 subtypes are also included in the analysis. Names of sequences obtained by us are in blue. In reference sequences, the subtype or CRF is indicated before the virus name. Only ultrafast bootstrap values ≥ 90% are shown. The scale indicates substitutions/site. *10BR_MG003 and 10BR_MG005 were originally identified as CRF72_BF1 (Pessôa et al., 2014b,^2016^), but analyses described in this study have reclassified them as CRF122_BF1.

In order to determine whether the F1_2 cluster was of uniform subtype or recombinant, we obtained NFLG sequences from three individuals from two cities through amplification from plasma RNA. A fourth NFLG sequence had been obtained previously from the virus culture supernatant (Sanchez et al., 2014). Preliminary analyses of the NFLG with Recombination Identification Program^2^ indicated that the genomes were BF1 recombinant. To determine whether they belonged to a known CRF, we constructed a phylogenetic tree in which genomes of all CRF_BFs were included. The tree showed that viruses of the F1_2 cluster grouped in a strongly supported clade with viruses classified as CRF72_BF1, with two of them, 10BR_MG003 and 10BR_MG005, being the most closely related to the viruses of the F1_2 cluster, and the other three, 10BR_MG002, 10BR_MG004, and 10BR_MG008, branching in a sister clade (Figure 2).

**FIGURE 3:**
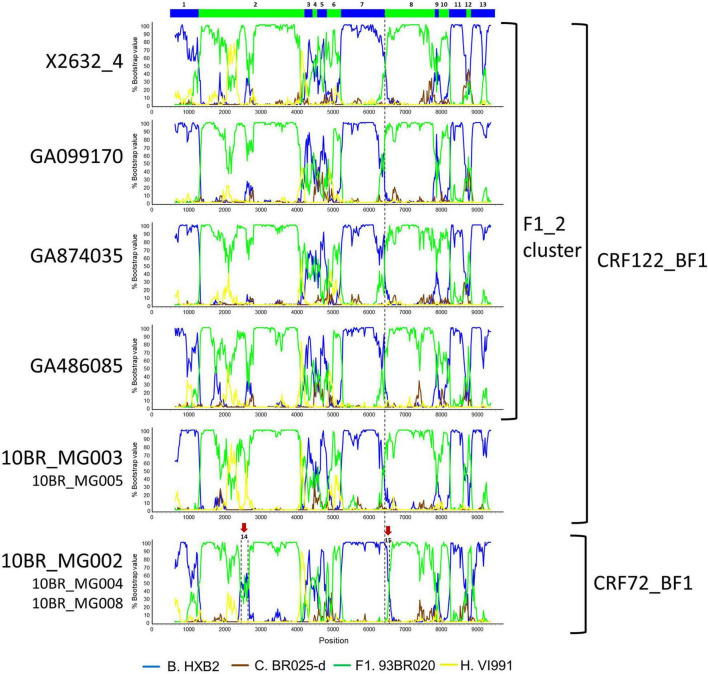
Bootscan analyses of NFLG sequences of viruses of the F1_2 cluster compared to those of viruses previously classified as CRF72_BF1. Bootscan plots of all four F1_2 viruses are shown, together with those of 10BR_MG003 and 10BR_MG002. The bootscan plot of 10BR_MG005 is almost identical to that of 10BR_MG003, and those of 10BR_MG004 and 10BR_MG008 are almost identical to that of 10BR_MG002, and are shown in Supplementary Figure 1. The horizontal axis represents the position in the HXB2 genome of the midpoint of a 250-nt window moving in 20-nt increments, and the vertical axis represents bootstrap values supporting clustering with subtype reference sequences. The vertical dashed lines indicate BF1 breakpoints differing between the CRF72_BF1 viruses 10BR_MG002, 10BR_MG004, and 10BR_MG008, on the one hand, and viruses of the F1_2 cluster and 10BR_MG003 and 10BR_MG005 (newly identified as CRF122_BF1), on the other. The bar on the top indicates the segments that were further analyzed with ML and Bayesian trees (Figure 4). Two genome segments that appear to group with different subtypes in 10BR_MG002, 10BR_MG004, and 10BR_MG008 relative to 10BR_MG003, 10BR_MG005, and the F1_2 viruses are signaled with arrows above the bootscan plot of 10BR_MG002 and were also analyzed *via* ML and Bayesian inference (Figure 4).

**FIGURE 4:**
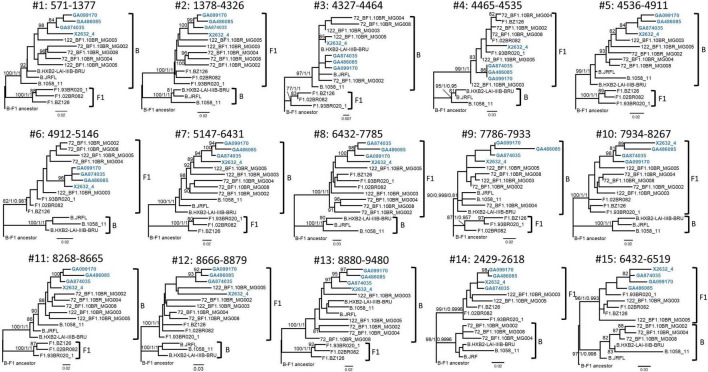
Phylogenetic trees of interbreakpoint genome segments of F1_2 viruses. Breakpoints were defined according to the bootscan analyses and to midpoints of transitions between subtype-discriminating nucleotides, here defined as those where the 75% consensus of subtype B and of the Brazilian variant of subsubtype F1 differ. HXB2 positions delimiting the analyzed segments and their numbers as indicated in Figure 3 are indicated on top of the trees. Sequence names of F1_2 viruses are in blue. Names of subtype reference sequences are preceded by the corresponding subtype name. Sequences of viruses previously classified as CRF72_BF1 were also included, with those reclassified in the present study as CRF122_BF1 (10BR_MG003 and 10BR_MG005) labeled with the new CRF designation. The trees are rooted with a reconstructed BF1 ancestor. Node supports for B and F1 clades are indicated, in this order, as ultrafast bootstrap value/aLRT SH–like support/posterior probability, which were obtained with IQ-Tree, PhyML, and MrBayes programs, respectively. For the other nodes, only ultrafast bootstrap values ≥ 80% are indicated. Trees #14 and #15 (segments 2429–2618 and 6432–6519, respectively) correspond to the segments indicated with arrows in Figure 3, where F1_2 viruses and 10BR_MG003 and 10BR_MG005, on the one hand, and 10BR_MG002, 10BR_MG004, and 10BR_MG008, on the other, appeared to differ in subtype affiliations in the bootscan analyses. The scales indicate substitutions/site.

**FIGURE 5:**
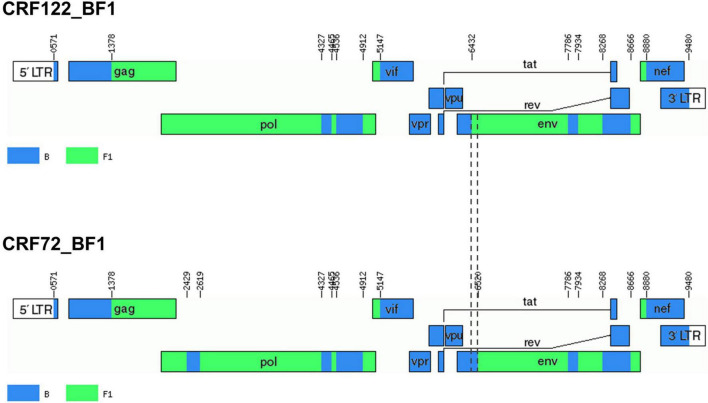
Mosaic structures of CRF72_BF1 and CRF122_BF1. Breakpoint positions are numbered as in the HXB2 genome. Vertical dashed lines indicate the BF1 breakpoint positions in the 5′ segment of gp120^env^ differing between CRF122_BF1 and CRF72_BF1. The drawing was made using the Recombinant HIV-1 Drawing Tool (https://www.hiv.lanl.gov/content/sequence/DRAW_CRF/recom_mapper.html).

Bootscan analyses of NFLG sequences showed that the viruses of the F1_2 cluster were BF1 recombinant, exhibiting mosaic structures fully coincident with those of 10BR_MG003 and 10BR_MG005, and slightly different from the three other viruses classified as CRF72_BF1 (Figure 3 and Supplementary Figure 1). The differences between these three viruses were observed in a short *pol* segment, around the protease–reverse transcriptase junction, where grouping with subtype references was discrepant, and in the 5′ segment of gp120, where the location of a BF1 breakpoint differed. The mosaic structures determined with bootscanning were confirmed by ML and Bayesian phylogenetic analyses of partial genome segments, which confirmed the coincidence of the mosaic structures of the four F1_2 viruses and the Brazilian 10BR_MG003 and 10BR_MG005 viruses and the subtype discrepancy in two genome segments (HXB2 positions 2429–2618 and 6432–6519) of these viruses with 10BR_MG002, 10BR_MG004, and 10BR_MG008 (Figure 4). These analyses, therefore, allowed determining that viruses of the identified Spanish BF1 cluster, together with the Brazilian viruses 10BR_MG003 and 10BR_MG005, previously classified as CRF72_BF1, belong to a CRF, which was designated CRF122_BF1, which is closely related to, but different from, the three other viruses previously classified as CRF72_BF1, 10BR_MG002, 10BR_MG004, and 10BR_MG008, whose original CRF designation is maintained. The mosaic structures of both CRFs, as inferred from bootscan analyses, ML and Bayesian phylogenetic trees of partial sequences, and examination of intersubtype transitions of subtype-discriminating nucleotides, are shown in Figure 5.

Three additional BF1 recombinant NFLGs, originally identified as unique recombinant forms, two from Brazil, 99UFRJ-2 (Thomson et al., 2004) and BREPM1029 (Sa-Filho et al., 2007), and one from Italy, IT_BF_PRIN_454 (Bruselles et al., 2009), in their published analyses, exhibited mosaic structures similar to CRF72_BF1 and CRF122_BF1. To determine whether they belonged to one of these CRFs, we constructed a phylogenetic tree with NFLG sequences including the three mentioned genomes, which showed that all of them grouped with CRF72_BF1 viruses (Supplementary Figure 2). Bootscan analyses showed mosaic structures of 99UFRJ-2 and IT_BF_PRIN_454 coincident with that of CRF72_BF1; however, the bootscan plot of BREPM1029 failed to show clustering with the subtype B references in the protease–RT junction (HXB2 positions 2429–2618) (Supplementary Figure 3). Examination of subtype-discriminating nucleotides suggested that the 2429–2618 segment was of subtype B, as in CRF72_BF1, in 99UFRJ-2 and IT_BF_PRIN_454, which was confirmed by phylogenetic analyses of this fragment (Supplementary Figure 4a). However, in BREPM1029, the subtype B fragment in the protease–RT junction appeared to be slightly shorter, located between HXB2 positions 2479 and 2618, which was confirmed by phylogenetic trees (Supplementary Figure 4b). Phylogenetic analyses also showed that in all three genomes the 6432–6519 segment in gp120 was of subtype B, as in CRF72_BF1 and unlike CRF122_BF1 (Supplementary Figure 4c). These results allowed to confidently classify 99UFRJ-2 and IT_BF_PRIN_454 as CRF72_BF1 viruses. As to BREPM1029, given its strong phylogenetic clustering with CRF72_BF1 references and its minimal difference in mosaic structure with CRF72_BF1, with a breakpoint displaced only around 50 nt relative to this CRF, it seems reasonable to also classify it as CRF72_BF1, although we cannot definitively discern whether its breakpoint displacement is due to a different recombination event or to mutations occurring near the CRF72_BF1 breakpoint.

### Differences in Amino Acid Residues

We analyzed amino acid residues in viral proteins differing between CRF72_BF1 and CRF122_BF1 viruses and conserved within each CRF. We found 10 such amino acid residues (Table 2). One of them is in position 89 of protease, where CRF72_BF1 has leucine, which is the subtype B consensus, while CRF122_BF1 has methionine, which is the F1 subsubtype consensus. Protease L89M substitution has been reported to contribute, together with other protease mutations, to resistance to some protease inhibitor drugs (Calazans et al., 2005; Marcelin et al., 2008; Wensing et al., 2019).

**TABLE 2:** Differences in amino acid residues between CRF72_BF1 and CRF122_BF1*.

		p17^gag^	PR		RT	IN	Vif	Tat	Rev	Vpu	gp120^env^
										
		61	61	89	399	84	151	68	22	80	69
B	HXB2	L	Q	L	E	I	A	S	L	D	D
CRF72_BF	10BR_MG002	I	Q	L	E	L	V	S	L	D	D
	10BR_MG004	I	Q	L	E	L	A	S	L	D	D
	10BR_MG008	I	Q	L	D	L	A	S	L	D	D
	99UFRJ-2	I/M	Q	L	E	L	A	P	L	D	D
	BREPM1029	L	N	L	E	L	A	S	L	D	D
	IT_PRIN_454	I	Q	L	E	L	A	S	L	D	D
CRF122_BF1	10BR_MG003	L	N	M	D	I	T	D	I	N	N
	10BR_MG005	L	N	M	D	I	T	D	I	N	N
	X2632_4	L	N	M	D	L	T	D	I	N	N
	GA099170	L	N	M	D	I	T	D	I	N	N
	GA486085	L/I	N	M	D	I/M	T	D	I	N	D
	GA874035	L	N	M	D	I	T	D	I	N	N

*Only amino acid residues conserved in at least five of six CRF72_BF1 viruses and in both Brazilian and at least three of four Spanish CRF122_BF1 viruses are included in the table.

### Antiretroviral Drug Resistance Mutations

Primary ARV drug resistance mutations were detected in two CRF122_BF1 viruses, both from Brazil, one (10BR_MG003, collected in 2010) with L90M protease inhibitor (PI) resistance mutation and the other (BR05SP503, collected in 2005) with D30N PI resistance mutation and M41L, D67N, M184V, and T215Y mutations of resistance to nucleoside RT inhibitors.

### Temporal and Geographic Estimation of CRF122_BF1 Origin

**FIGURE 6:**
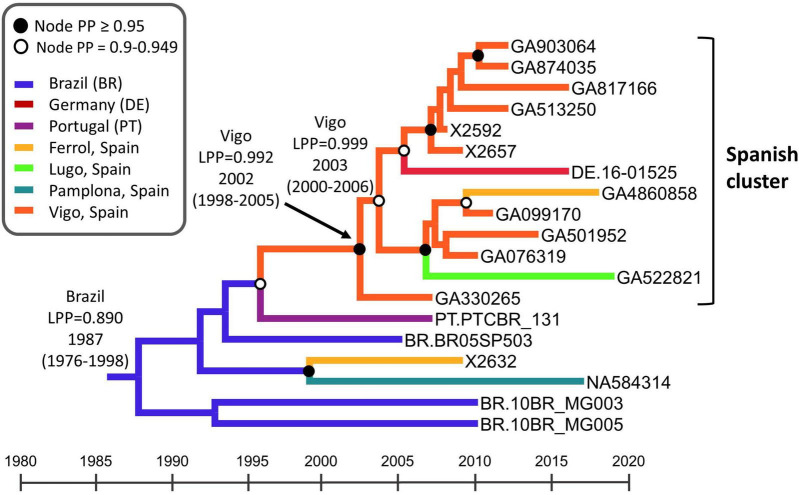
Maximum clade credibility tree of CRF122_BF Pr–RT sequences. Branch colors indicate, for terminal branches, the place of sample collection and, for internal branches, the most probable location of the subtending node, according to the legend on the upper left. Nodes supported by PP ≥ 0.95 and PP 0.9–0.949 are indicated with filled and unfilled circles, respectively. The most probable locations at the root of the tree and at the node of the Spanish cluster are indicated, together with the PPs supporting each location (LPPs) and the year estimated for the MRCAs (mean value, with 95% HPD interval in parentheses). The scale under the tree represents calendar years.

To estimate the time and place of origin of CRF122_BF1, Pr–RT sequences were analyzed with the Bayesian coalescent method implemented in BEAST 1.10.4. Prior to this analysis, we performed TempEst analyses to determine whether there was an adequate temporal signal in the dataset. We found that the temporal signal, assessed by the correlation between root-to-tip distance and time, increased by masking the positions of codons with drug resistance mutations in any of the sequences (*r*^2^ = 0.5265; Supplementary Figure 5). Therefore, the BEAST analysis was performed with a sequence alignment where these codons had been removed. In this analysis, the substitution rate was estimated at 1.829 × 10^–3^ subs/site/year (95% HPD, 1.118 × 10^–3^–2.542 × 10^–3^ subs/site/year) and the time of the MRCA of CRF122_BF1 was estimated around 1987 (95% HPD, 1976–1998), with its most probable location being Brazil (location PP = 0.89) (Figure 6). The introduction of CRF122_BF1 in Spain (according to the MRCA of the Spanish cluster) was estimated in the Galician city of Vigo (location PP = 0.992) around 2002 (95% HPD, 1998–2005).

## Discussion

The results presented here indicate that the five Brazilian viruses previously classified as CRF72_BF1 actually belong to two closely related CRFs, one of which is circulating in Spain. Consequently, the CRF comprising two Brazilian viruses previously classified as CRF72_BF1 and the four Spanish viruses with coincident mosaic structures is given a new designation, CRF122_BF1, while the three other Brazilian viruses previously classified as CRF72_BF1 keep their original designation. Three other BF1 viruses analyzed in NFLGs originally classified as URFs, two from Brazil and one from Italy, were also classified on the basis of phylogenetic and bootscan analyses as CRF72_BF1. The close relationship between CRF122_BF1 and CRF72_BF1 is one more example of closely related CRFs, with precedents in South America. Other examples are CRF12_BF, CRF17_BF, and CRF89_BF (and more distantly related, CRF38_BF and CRF44_BF) (Delgado et al., 2021); CRF28 and CRF29_BF (De Sá Filho et al., 2006); and CRF70_BF and CRF71_BF (Pessôa et al., 2014a).

Failure to realize that the five viruses previously identified as CRF72_BF1 represent two different CRFs may derive from the short segments in which both CRFs differ in subtypes. These differences may be missed if bootscan analyses are performed using window widths much greater than the length of the recombinant fragment. We have also noticed that jpHMM (Schultz et al., 2009), used in a previous study to analyze CRF72_BF1 genomes (Pessôa et al., 2016), often fails to detect short recombinant segments (Delgado et al., 2021).

Given the close relationship and partial coincidence in mosaic structures of CRF72_BF1 and CRF122_BF1, we propose that they are members of a CRF family, similar to the CRF family of BF1 recombinant viruses from South America comprising CRFs numbers 12, 17, 38, 44, and 89 (Delgado et al., 2021). The grouping of some closely related HIV-1 recombinants derived from a common recombinant ancestor in families was proposed by Thomson and Nájera (2005) and Zhang et al. (2010). The proposed families of CRFs with close phylogenetic relations, shared parental strains, and partially coincident breakpoints are indicated in the phylogenetic tree shown in Supplementary Figure 6.

It is interesting to note that, in the Pr–RT tree, viruses from the Spanish CRF122_BF1 (“F1_2”) cluster fail to group with the Brazilian CRF122_BF1 viruses. A similar phenomenon is observed with CRF66, CRF70, and CRF71_BF1 references, that fail to group in distinct clades with other references of the same CRF. This may be due to the relatively short length and high sequence conservation of this segment, together with the fact that 35 references from the Brazilian F1 strain or from CRF_BFs derived from it are included in the tree. This shows that exclusive phylogenetic analysis of Pr–RT may not be sufficient to phylogenetically classify an F1 sequence of the Brazilian strain as belonging or not to a given CRF_BF.

The estimated origin of CRF122_BF1 around 1987 is consistent with the estimated origin of the Brazilian F1 strain (around 1977) (Bello et al., 2007) and similar to those of other South American CRF_BFs (CRF12, CRF28/29, CRF38, CRF89, and CRF90) reported in the literature (Bello et al., 2010; Ristic et al., 2011; Reis et al., 2017; Delgado et al., 2021) but younger than some other estimates for CRF12_BF in the 1970s (Dilernia et al., 2011; Delgado et al., 2021) and older than the estimates for CRF99_BF, around 1993 (Reis et al., 2017).

The correct classification of HIV-1 genetic forms is important, since even relatively minor genetic differences in viral genomes may result in important biological differences. Examples in HIV-1 are frequent CXCR4 coreceptor usage in CRF14_BG, which is associated with only four amino acid residues in the Env V3 loop (Pérez-Álvarez et al., 2014), all or most of which are absent in viruses of the closely related CRF73_BG (Fernández-García et al., 2016), which has a very similar mosaic structure, and differences in pathogenic potential or therapeutic response associated with clusters within HIV-1 CRF01_AE (Song et al., 2019) and F1 subsubtype (Cid-Silva et al., 2018). Here, we show that CRF122_BF1, but not CRF72_BF1, has the protease L89M substitution, that has been reported to contribute, together with other protease mutations, to resistance to tipranavir/ritonavir (Marcelin et al., 2008) and, within an F1 subsubtype background, to other protease inhibitor drugs (Calazans et al., 2005).

CRF122_BF1 represents one more example of a CRF of South American ancestry first identified in Western Europe. Others are CRF42_BF (Struck et al., 2015), CRF47_BF (Fernández-García et al., 2010), CRF60_BC (Simonetti et al., 2014), CRF66_BF (Bacqué et al., 2021), and CRF89_BF (Delgado et al., 2021). This may derive from the increasing migratory flows from South America to Europe and from the relatively low number of HIV-1 sequences available in some South American countries (Bacqué et al., 2021). Therefore, HIV-1 molecular epidemiological studies in Europe may contribute to a better knowledge of the HIV-1 epidemics in South America.

In summary, we show that viruses of a BF1 recombinant cluster of Brazilian ancestry circulating in Spain exhibit a mosaic structure that is fully coincident with that of two Brazilian viruses previously classified as CRF72_BF1 and is highly similar, but not identical, to that of three other Brazilian viruses also classified as CRF72_BF1. Therefore, we propose a new CRF designation, CRF122_BF1, for the viruses of the Spanish cluster and the two Brazilian viruses with coincident structures, which together with CRF72_BF1 would constitute a CRF family. The accurate genetic characterization of HIV-1 variants is important to determine their associated biological features and to track their epidemic spread.

## Data Availability Statement

The datasets presented in this study can be found in online repositories. Newly derived sequences are deposited in GenBank under accessions OL982311–OL982317 and OL982320–OL982323. Phylogenetic trees and alignments used for their construction have been deposited in TreeBase, with accession URL http://purl.org/phylo/treebase/phylows/study/TB2:S29595.

## Ethics Statement

The studies involving human participants were reviewed and approved by Committee of Research Ethics of Instituto de Salud Carlos III, Majadahonda, Madrid, Spain. Written informed consent for participation was not required for this study in accordance with the national legislation and the institutional requirements.

## Author Contributions

MT and ED conceived the study and supervised the experimental work. JC-G, ED, and MT processed the sequences and performed phylogenetic and phylodynamic analyses. HG performed data curation. JC-G, SB, MS, and EG-B performed experimental work. AO, JC, CM, AM, PO, MG, and CE obtained the samples and epidemiological data from patients. MT, JC-G, ED, and HG wrote the manuscript. All authors read and approved the manuscript.

## Conflict of Interest

The authors declare that the research was conducted in the absence of any commercial or financial relationships that could be construed as a potential conflict of interest.

## Publisher’s Note

All claims expressed in this article are solely those of the authors and do not necessarily represent those of their affiliated organizations, or those of the publisher, the editors and the reviewers. Any product that may be evaluated in this article, or claim that may be made by its manufacturer, is not guaranteed or endorsed by the publisher.

## Footnotes

1
http://tree.bio.ed.ac.uk/software/figtree/

2
https://www.hiv.lanl.gov/content/sequence/RIP/RIP.html
